# How Organisms Gained Causal Independence and How It Might Be Quantified

**DOI:** 10.3390/biology7030038

**Published:** 2018-06-29

**Authors:** Keith Douglas Farnsworth

**Affiliations:** School of Biological Sciences, Queen’s University Belfast, 97 Lisburn Road, Belfast BT97BL, UK; k.farnsworth@qub.ac.uk

**Keywords:** autonomy, IIT, causation, autopoiesis, cognition, action-selection, agency, free will, consciousness

## Abstract

Two broad features are jointly necessary for autonomous agency: organisational closure and the embodiment of an objective-function providing a ‘goal’: so far only organisms demonstrate both. Organisational closure has been studied (mostly in abstract), especially as cell autopoiesis and the cybernetic principles of autonomy, but the role of an internalised ‘goal’ and how it is instantiated by cell signalling and the functioning of nervous systems has received less attention. Here I add some biological ‘flesh’ to the cybernetic theory and trace the evolutionary development of step-changes in autonomy: (1) homeostasis of organisationally closed systems; (2) perception-action systems; (3) action selection systems; (4) cognitive systems; (5) memory supporting a self-model able to anticipate and evaluate actions and consequences. Each stage is characterised by the number of nested goal-directed control-loops embodied by the organism, summarised as will-nestedness N. Organism tegument, receptor/transducer system, mechanisms of cellular and whole-organism re-programming and organisational integration, all contribute to causal independence. Conclusion: organisms are cybernetic phenomena whose identity is created by the information structure of the highest level of causal closure (maximum N), which has increased through evolution, leading to increased causal independence, which might be quantifiable by ‘Integrated Information Theory’ measures.

## 1. Introduction—Life As a Challenge to Physics

It is generally accepted that life is an elaboration of chemistry [1], with the implication that at a molecular level, living processes are seamlessly a part of the non-living cycles of Earth chemistry and ultimately of the universe. Scientists long ago rejected the idea of a ‘life-force’ giving special properties (e.g., of self-determination) to the living [2]. But plants contort to follow light, bacteria perform chemotaxis, cells collaborate in achieving collective actions (as in multicellular organism development) and animals appear to choose their actions from a repertoire of internally generated behaviours. All these seem to be goal directed behaviours, which implies that the cause of their actions is, at least in part, derived from within the organism, i.e., not determined solely by the chain of cause and effect following the immutable laws of physics in the world around them. This apparent causal autonomy is a long standing puzzle that is not completely solved by saying that such behaviours must have come about through evolution. That is because—more fundamentally—our understanding of the physical universe of matter, forces and chemistry, precludes any spontaneous cause, other than from random events at the microscopic level (e.g., from thermal noise or quantum fluctuation). Goal-directed behaviour in living systems (biological agency) is definitively organised, hence the opposite of random, and therefore demands another scientific explanation [3] (the universe is full of random events, but if an action is caused by one of these, it cannot properly be attributed to the agent within which it occurred). Additionally, according to Walker and Davies [4], the transition “from matter to life” when the first true organisms appeared (uniquely, since all new organisms and cells subsequently have come into being through the division of a common ancestor), raises the “hard problem of life” which is “how ‘information’ can affect the world” (see next section and Appendix A for definitions of information-related terms). In this, they recognise that biological agency is fundamentally a phenomenon of matter (seemingly) obeying the control of ‘information’, despite the latter having no momentum, force, or energy. The idea that “information can be causal” is superficially familiar (we believe that our own actions are partly determined by information), but more deeply it contradicts conventional physics—in which only forces can have agency—with the surprising result that the study of living systems has come to pose an important puzzle for modern physics [4]. Put simply, the most unique feature of life— autonomous agency—seems incompatible with physics as currently constituted. Some have suggested this may indicate an as yet undiscovered law of physics [4]. Here I hope to make a more modest case for finding an explanation founded in conventional physics and a constitutive information theory (separately from Shannon’s communications theory), a part of modern cybernetics (abstract control theory), making use of the ‘Integrated Information Theory’ (IIT) of Tononi and colleagues [5,6,7,8,9] (which is a recent development in information theory and cybernetics intended for understanding consciousness), all illustrated through biological example.

Given the insight that ‘information’ and its processing are fundamental to life, an obvious way to understand the peculiar properties of life is through information-theory and cybernetetics, famously initiated by Schrödinger [10] and Wiener [11] and now a rapidly developing perspective in biology [12,13,14,15,16]. One thread of this effort is based on earlier insights concerning the organisation and implied control systems characterising living processes [3,17,18,19,20,21,22,23,24,25,26]. A set of key concepts have emerged from this work: constitutive autonomy [27], downward causation by information control [28] in the context of autocatalytic sets [29], modular hierarchical organisation [30] and biological computation [31] (see also [32]).

Here I will emphasise the *embodying* of those cybernetic systems by the material of biological systems–networks of chemical interactions arranged in space and time– such that ‘living’ is continuous with all physical systems. But I also emphasise that the identity and cause of the behaviour of organisms is derived from (and in a meaningful sense *is*) the information embodied, rather than the material embodying that information. Life is essentially the result of the hierarchical nesting of causal networks [33,34] in which some degree of top-down control is created at each hierarchical level [12]. The information instantiated by these networks is primarily embodied in biomolecular interactions (e.g., the growth factor receptor interaction network described by Koseska and Bastiaens [35]), which combine to form functional systems with the properties of determinate machines (Finite State Automata—FSA) as illustrated by the classic case of the *Escherichia coli* chemotaxis system and explicitly identified in plant physiology [36]). These FSAs are elaborated to include simple learning and action selection in protozoa for which *Stentor* species provide an archetype. Such single celled organisms already display the essential autonomous control systems for free self-generated behaviour. The advent of nervous systems, through differentiation of specialist neuronal cells in certain early metazoans (of which *Cnydarians* are representative) and elaboration, through Bilateria, of primitive brains (exemplified by the neural network of *Caenorhabditis elegans*) and on through the chordates, vertebrates, mammals and finally great apes and humans, therefore are all adjuncts: presumably evolved to better manage the increasingly complex tasks of multicellular, multi-organ, multi-limb, and finally, social integration. A parallel development of nervous systems proceeded among the protostomes-arthropods (including social insects) and molluscs (including the famously ‘clever’ octopi), for which Prescott [37] gives a more detailed account. The cybernetic approach is this: the whole behavioural system of, e.g., *Stentor* could be represented in an algorithm, which would amount to describing it by a particular representation of information. This could then (hypothetically) be embodied in logic circuits composed of transistors and other silicon-junction technology because an algorithm is *multiply realisable*, i.e., independent of its material substrate. The circuitry would then have embodied the information contained in the algorithm and in the same way, the molecular interaction networks of living *Stentor* embody the same information. We may take the networks or their electronic equivalent and quantify (see below) the information embodied by the level of organisation that, in a cybernetic sense *is* the *Stentor*. (Note, a related idea is debated by philosophers, especially in the context of the ’neural replacement thought experiment’ of Pylyshyn [38] in response to Searle [39]).

In every case we study, the biochemical control circuitry of a cell, or nervous system of a multi-cellular organism, has the role of integration of the organism’s parts into a whole and sometimes the further integration of this whole into a higher level of integration—a society. This integration is one of information flows and control systems and each of these shifts in complexity appears to be associated with the emergence of a higher organisational level of integration, so that taken together they form a nested hierarchy. These levels can be identified and their information ‘richness’ may be quantifiable using measures such as the integrated information metric of IIT [5], indeed this was developed to quantitatively examine the hierarchical structure of living (originally neural) control systems and to elucidate the relation between physical embodiment and the emergence of ‘consciousness’. Other than the social level, the highest level in the control hierarchy is identified with the organism itself, so attributes such as ‘intention’ and ‘selfhood’ properly belong to the emergent integrating information at the whole-organism level. Thus, when we consider an autonomous agent, we (implicitly) refer to this integrating information which is embodied (in the case of life) by chemical interaction networks. The logical conclusion of this line of thinking is that the “I” in Descartes’ famous deduction “I think therefore I am” is the integrating information embodied at the level of an individual whole human. Descartes thought “I” to be a disembodied mind; we now think of it as a necessarily embodied information complex and if this is a difficult concept, bear in mind that almost all the molecules of the body are regularly replaced, while the person remains who they are.

### Information Concepts for Biology

There are two levels of abstraction in which we can understand the components of biological agency: the more abstract and general cybernetic theory and the more biologically material level, concerning signal pathways and nerves and their functions of cognition, action selection and behavioural integration. Both are needed to account for biological agency. Extended definitions for relevant information terminology are provided in the Appendix A (though these are not necessarily settled and agreed, they form a self-consistent set of working definitions for the presented arguments). Here in the main text, I will briefly introduce the concepts and then concentrate on a short introduction to IIT as a method for quantifying agency, since this will inform all that follows.

I use two different concepts of *information*. The conventional communications theory concept, mostly attributed to the work of Claude Shannon, expresses the reduction in the range of possibilities brought about from the addition of information and is used extensively in bioinformatics. The other, essentially Aristotelean concept is suggested by the word itself: *in*ternalised *form*ation, i.e., the embodiment by the form of a thing (its shape, structure and composition) of all that is needed to know to reproduce it. The communications meaning of information is necessarily a relational quantity describing the change in a ‘receiver’ system resulting from a transaction with a ‘sender’, for example the transfer of a message or of genetic information from one generation to the next and it is quantified by the reduction in the number of possible inputs (e.g., messages or genetic instructions), starting with all possible (complete ignorance) and ending (at best) with only the one received (perfect information). The number of possible inputs is calculated from the number of different ‘symbols’ (e.g., letters in an alphabet or nucleotides) and the number of ways they can be arranged in a message: this idea is captured by the quantity *entropy*. A little more broadly, we can think of entropy as the number of possible ways to arrange an ensemble of objects and think of selecting (filtering out) a sub-set of those ways as providing information. If the objects are components of a system (e.g., molecules, organisms or machine parts) and the way of arranging them is a specific one that forms e.g., a cell, an ecological community, or a motorbike, then that assemblage has a (functional) form (a pattern) which embodies (Aristotelean) information equivalent to the amount of selection needed to identify it from all other possible assemblies. This embodied pattern-information is greater if the assembly of components is larger and more varied (as in biodiversity) and the size and variety of the ensemble of components is quantified by the entropy of the ensemble. For any ensemble there are many ways to arrange the components, but usually very few resulting in a form that holds together and performs some function, i.e., has a role in a wider context. In such a case, the pattern information of the particular form is *functional information* and it is embodied in both the relations among the components (their placing relative to one another) and the possible interactions among them. The set of inter-relations among the components is an abstract, purely organisational, phenomenon that *transcends* the material composition of the components, in the sense that we can think of it without referring to them and sometimes can translate it from one medium to another, e.g., making a computer using water pipes and mechanical valves, or more subtly, a cell forming a signalling pathway having a choice of proteins. This functional information embodied among interactions is what I term a *transcendent complex*. Among possible transcendent complexes, some have the special property of being *organisationally closed*.

Closure is a mathematical concept (defined more formally in the Appendix A) with a broad but precise meaning for an operation on a set, roughly as follows: if for some mathematically valid thing you can do (an operation) to a particular set of objects A (e.g., if A is a collection of numbers and the operation is addition), the result is always a set of objects that are all members of A, then A is said to be closed with respect to the operation. This general idea is applied in cybernetics, especially when the operation is one relating one object to another, e.g., if A is a set of chemical reagents, R may be the set of reactions that could take place among them and these reactions are obviously relational operations among the members of A. Because this kind of closure is about relations it is termed *relational closure*. A system with *organisational closure* is a special case of this: it is one in which the form of the whole is produced by the interactions among its component parts alone, without any exogenous intervention, i.e., it is self-organising and the usual example is a vortex (e.g., a whirlpool or cyclone). Systems with organisational closure are self-sustaining organisations (the organisation is information embodied as a transcendent complex) that only need an appropriate source of energy to maintain their form. Taking a step closer to life, in an autocatalytic set of chemical reagents the members of the set operate on one another to produce one another and thereby also to produce the system of interactions that perform the autocatalytic operations. This is clearly important in establishing autonomy, though an autocatalytic set alone cannot sustain itself without also involving a containment boundary. A system which creates its own transcendent complex, and also all the component parts from which it itself is made, and can sustain itself, is an *autopoietic* system, though this does not imply the atoms themselves are made by it (normally it means simpler—raw material—molecules are selected from the environment and assembled into functional molecular components). This underlines the point that organisational closure still leaves the system open to material and energy flows. Obviously, in the case of autopoiesis, the function of the transcendent complex is the making (and maintenance) of the form. In living systems, which just as obviously have to adapt to and use their environment, this cannot be maintained in isolation. Living things not only need to be organisationally closed, but also must develop *informational closure*: they need to *embody* information about their environment that enables them to *autonomously* respond in a functionally appropriate way towards it. This involves incorporating (literally) information gained from sensing the environment. This embodiment may be in the design of the organism (the result of natural selection, which is the filtering of all possible forms to leave only the adapted form—hence information by virtue of reducing the entropy of the range of designs), or the establishment of a chemically or neurologically mediated memory. In scientifically evaluating the autonomy of organisms, it is obviously going to be important to quantify the information incorporated in environmentally informed autopoietic systems of this kind.

IIT was developed to explain the phenomenon of conscious experience in terms of underlying (generating) physical systems, the basic premise being that it must be the product of a physical (neuronal) system having the same broad properties as those of conscious experiences: that they exist, are definite, structured, differentiated and unified [5]. At its root, IIT claims that “at the fundamental level, consciousness is integrated information” (Tononi [6], p. 217). As a radical idea in a contentious field of study for which we still have very little scientific evidence and understanding, it has unsurprisingly met some criticism and opposition (e.g., Cerullo [40]). Whether or not the concept of integrated information may eventually explain consciousness, it is already useful for quantifying the ‘wholeness’ of causal networks, such as systems containing ‘circular causality’ [2], which are characteristic of life and fundamental to the emergence of autonomy (the original idea of circular causality came from Fuchs [41]—see Appendix A).

For the purpose of the present work, there is no need to accept the inference of IIT for consciousness, we can take integrated information as a quantitative concept that may be useful in describing the properties necessary for autonomous agency. As the name suggests, integrated information (hereafter φ) is another information metric, though a complicated one. In the language of IIT: φ is a measure of the irreducibility of the cause-effect repertoire specified by a ‘mechanism’ compared to its minimum information partition (MIP). It is “Information that is generated by a mechanism above and beyond the information generated by its (minimal) parts. φ measures the integration or irreducibility of mechanisms (integration at the mechanism level)” (Oizumi et al. [7], pp. 4–5). It might be summarised as the information embodied by a system over and above information embodied by its parts taken separately, but crucially this is information that embodies functional relations between the parts. Specifically, functional here means that the set of possible states (and the flow from one state to another) in the system is constrained to a small subset of all the states available (thus meeting the general entropy definition of information as a filter on the range of possibilities). For this reason, what φ measures has been referred to as a ‘ causal architecture’. What is meant by causality in IIT is the necessity (obligation by the constraint of functional information) of a state (for any part or whole of a system), given the state of any part or whole of the system. This ‘necessity’ is imposed by the definition of the parts (most clearly if they are logic operators such as AND and OR gates, where a ‘truth table’ defines the necessary effects of a given state). In other words, given a set of interconnected parts, each *defined* by their response to all possible inputs, and a complete description of their present states, their next state is fully defined for all of them and therefore also for the system as a whole: the present state is the *cause* and the next state is the *effect*. Specifying the present states obviously constrains the range of possible outcomes and this clearly constitutes information (in the classic Shannon sense of reduced uncertainty). Defining the repertoire of all possible responses of all the parts (as in a truth table) also constrains the range of possible outcomes and therefore also constitutes information. Indeed, it seems that with both the truth table and the present state, we have all the information present in the system, but φ represents *additional* system-level functional information, over and above that embodied by the system’s parts. Where is it embodied? The answer must be in the cybernetic architecture of connections among the parts: the wiring of the logic circuitry, or the relations among components in an automaton. In a biological system, these are the specific chemically mediated relations (which depend on timing) among components such as specific protein molecules. φ therefore measures the contribution of the system, over and above it component parts, to embodying the functional pattern information (Appendix A). It is therefore the pattern information of the ‘transcendent complex’ (ref. [13]—Appendix A) created by the cybernetic architecture of the system. This is why integrated information is potentially useful in understanding autonomy: it quantifies the pattern information of an irreducible transcendent complex, giving us (for the first time) empirical access to the cybernetic property of ‘relational closure’ (see Appendix A), which is closely associated with autonomy.

IIT rests on the conjecture that the organisational scale at which consciousness emerges is that which shows the greatest integrated information (the network scale for which φ is greatest), which is why it includes detailed methods for quantifying the relationship between φ and organisational scale. Various worked examples reveal astonishing complexity and richness in the causal structure of some algorithmically simple structures (Albantakis and Tononi [42] provide an example). But the procedure for calculating φ based on its definition for discrete Markovian (meaning that the next state depends only on the present state) systems is very cumbersome and limited [43]. More practical estimators have been developed in response by Balduzzi and Tononi [44] and further by Barrett and Seth [43], who offered methods to estimate φ from a time-series of states (dynamic behaviour) of a network that may be either continuous or discrete and is not restricted to be Markovian. This has opened the way to quantifying the extent to which biological systems embody more functional information than is to be found in their component parts.

The method has already been applied to the 277–neuron somatic network of *C. elegans* by Antonopoulos et al. [45]. These authors used established network analysis methods to identify six sub-graphs (partial networks, termed ‘communities’) within the *C. elegans* somatic network and modelled individual neuron behaviour with a well established continuous-time system of differential equations (so continuous and analogue), linked by one of two dynamic connection types: strong and fast for intra-community, weaker and slower for inter-community connections (representing electro-chemical and hormonal systems respectively). Their model produced a convincingly realistic simulation of the dynamic behaviour expected of a *C. elegans* neural network, including synchronisation within and among neural communities (as coupling strength among neurons was varied). Their calculations of φ from time-series of two different measures of neural activity showed φ>0 across a wide range of neuron model parameters and positively correlated with synchronisation as coupling strength was varied. Antonopoulos et al. [45] concluded that “for particular coupling strengths, the *C. elegans* BDN [brain dynamic network] is able to generate more information than the sum of its constituent parts” and they repeated the IIT interpretation that the higher φ>0, the higher the “level of consciousness”. This, though, is still a contentious definition of consciousness: it is more secure to claim that the greater φ (calculated from time-series data), the more causal power (Appendix A) can be attributed to the transcendent complex embodied by the network of interacting parts (neurons in this case).

The idea of information as a constraint (limiting the range of possibilities) is central to IIT, for which the state of elements within a system at time *t* may (at least partially, allowing for e.g., some randomness) determine those at t+k, therefore in turn being (at least partially) determined by the state at t−k, where *k* is a notional time-step. The system sets the criteria for how it responds to any given external information at any given moment and embeds those criteria in the physical structure of its own (biochemical, genetic or neural) networks. This cybernetic (information domain) model is natural, given the original purpose of IIT in developing an understanding of how networks of neurons construct ‘consciousness’ [6]. It is easily generalised to any discrete-state system (e.g., embodied by protein conformations), including the cell cycle as a discrete-state system (see e.g., Marshall et al. [46] for a relevant example). But it is crucial to understand this as an abstract model where cause and effect are implicit and the real (in the physical domain) cause for an effect is the vector sum of physical forces acting on an ensemble (Appendix A) of material particles. An explanation for agency must therefore establish a reliable (i.e., systematically observed and materially founded) link between physical forces as they are arranged in time and space and the changes in state of physical systems. In biological systems, the most relevant forces are the electrostatic interactions among protein molecule ‘surfaces’ that combine in protein interaction networks. As well as reaction kinetics, the topological (in practice, spatial) arrangement of these networks determines the cause-effect relations, as illustrated by the examples of Koseska and Bastiaens [35] where realistic biochemistry connected in different topologies results in rich and diverse dynamics.

## 2. How the Autonomy of Organisms Is Generated

The elements of autonomy in organisms will now be highlighted. These are a physical boundary and transducer system, cognition and learning, action selection and a nested structure of goal-directed downward causation systems. Examining each will lead through proposed stages in the ‘informational evolution’ of life on earth (analogous to the ‘energy expansions of evolution’ [47]), each stage being differentiated by the levels of information processing that can be exploited by organisms. I aim to organise these ideas into a coherent explanation of biological agency which accounts for the appearance of ‘free-will’, among at least some organisms, in terms of our existing understanding and compatible with biological observations. But first I need to clarify what I mean by agency and free-will, explaining how I see it not as an all-or-nothing attribute, but as an ordered set of capacities that reflect the organisational hierarchy of organisms.

### 2.1. Agency and Free-Will

The implication of agency is that a system to which it is attributed acts in a way that is systematic (not random) and such that the state (and maybe history of states) of the system (agent) is one of the determinants of the system’s behaviour. More specifically, the next state of the system is not random, not wholly determined by exogenous control, nor intrinsic to its structure (as in clockwork), but is at least partly determined by its present and (optionally) one or more of its previous states. The proximate cause of an action taken by an agent with agency is identified as its ‘will’. This proximate cause is not merely mechanism [48], it is the result of *information with causal power* rather than just deterministic *effective cause* (Appendix A).

Agency is defined here (following Farnsworth [14] definition of ‘free will’) as the condition in which all of the following are jointly true:

- D1: there exists a definite entity to which agency may (or may not) be attributed;
- D2: there are viable alternative actions for the entity to select from;
- D3: it is not constrained in the exercising of two or more of the alternatives;
- D4: its ‘will’ is generated by non-random process internal to it;
- D5: in similar circumstances, it may act otherwise according to a different internally generated ‘will’.

### 2.2. Will-Nestedness

The question addressed here is whether agency is a binary property of an autopoietic system, or a progressive capacity that migh be quantifiable. Autopoiesis is the process of self-making together with self maintaining; this concept being developed to understand the essence of ‘life as a process’ by Varela et al. [25]. If we abstract an autopoietic process from its material (molecular) basis, we are left with a *multiply realisable* cybernetic construct which specifies the functions required to achieve autopoiesis in potentially any medium. This functional specification is the algorithm for expressing autopoiesis at some (unspecified) level of organisation. It is to be found in every living cell, but also at the level of a whole multicellular organism, and it is hypothetically possible at organisational levels below that of organism (e.g., in mutually catalytic molecular sets [29,49,50,51] and, more speculatively, in the higher level of ecological systems [52,53]). In a model, or for an artificial life system, engineers may explicitly write the algorithm of autopoiesis, but, we might ask, where exactly is it within natural living systems and how did it get there? The answer seems to be that it is embodied in the network of parts of the system: the structure of the parts and their interactions and since, by definition this network was self-constructed, it is causally autonomous and therefore includes information which it is responsible for creating and maintaining. Self-maintenance in particular requires a set of homeostatic controls, each needing a set-point, which constitutes genuinely new information being embodied within the system [13]. When, for example, an organism osmoregulates, it is determining for itself an aspect of its internal conditions: an act which represents a first (superficial) level of cybernetic autopoiesis. If it additionally determines the value of that set-point for osmoregulation, by itself and according to a higher goal, then it displays a second (nested) level of self determination. In [14], I introduced the concept of ‘will-nestedness’ N to describe the depth of cybernetic autopoiesis of an agent. N counts the number of levels of causal power exercised over a system, *from within the agent as a whole*, the Nth level being the highest-level internal cause of its actions (thus enabling the conditions D4 and D5 above to be met). Will-nestedness is proposed as a way to understand how potential for ‘free will’ is an ordinal property of a system, rather than an all-or-nothing (binary) capacity, as it has traditionally been treated in the philosophical literature.

The logic of the idea is as follows. First, all non-random actions (causes of a change of state in a system) match changes in the information that are embodied by the matter and energy of the universe through their composition and arrangement in space and time, including that of the system itself. Forces are effective cause, but their direction, time and magnitude of effect depend on the arrangement of matter and energy in time and space, so at least in this sense, the embodied information has causal power.

Second, there exist material systems that are organised such that cause-effect relations (control circuitry) are in *transitive closure* (Appendix A), so that the state of every part depends on the state (and/or previous states) of every other part. This means that for any system having transitive closure, the cause of its next state must be determined by information embodied within its structure. This constitutes a first level of autonomy, setting N=1.

Third, it is understood (e.g., from Steel et al. [51]) that at least autopoietic systems have this property and that living organisms are the major example of autopoietic systems known to-date. Hence, no living system has N<1.

Fourth, it may be that such a system had created at least one piece of its own embodied causal information (information being created by a process of filtering of data, i.e., mutual correlation). The primary example of this process is learning, the simplest example being the learning of a suitable set point for a homeostatic control system. Because this constitutes the self-determination (‘will’) of a system over its own behaviour (even in the small respect of setting the set-point of homeostasis), in such a case, N>1.

Fifth, there is no impediment, in principle, to this nesting of top-down causation. For example, the information which created the embodied causal information that determined the set-point may itself have been created by and within the information structure of the system. As autopoietic systems, organisms can have, in principle, an unlimited level of will-nestedness. However, we shall see that to achieve N>2, special features, such as memory, action selection and an internal model of the self are progressively needed and broadly these are all supported by neural control systems, hence the evolution of nervous systems has served to extend the will-nestedness of organisms beyond a value of 2.

To illustrate the concept of will-nestedness, we may ask: “Do bacteria decide for themselves what to do?” ‘Themselves’ is well defined since bacteria are autopoietic, hence there exists a cybernetic system that integrates the bacterium into a coherent whole with a cybernetic boundary. But exactly what does it mean to ‘decide’? Does anything ‘decide’, or is it just following the universal chain of cause and effect? The answer is that if what an agent does next is contingent upon any information that it embodies internally, then it at least decides for itself in a superficial sense. That is the sense in which the next state of the agent can only be correctly predicted by taking account of information embodied within the agent itself. This is the case of the set-point of a homeostatic system for which N=1. If, further, the embodied information was created (instantiated) by the agent alone, then its next state is (partly) determined by information that in turn resulted from information embodied within the agent: N=2. If an agent’s actions (state changes) are entirely determined by exogenous changes, N=0. We understand that for a bacterium, N≥1 because it is autopoietic and more broadly, every part of its composition is dependent on every other part, both materially and in cybernetic terms (the so-called cellular operating system is organisationally closed). However, since every state-change of the living bacterium results from its molecular-level structure that was *evolved* by natural selection, the principles by which its causal information were determined lie beyond and before its making: specifically it *inherited* them from its parent, so N≤2. A closer examination (using the well described chemotaxis system of (e.g.,) *E. coli*) will shortly show that N=1 in such bactertia. Finally, note that will-nestedness is not the same as hierarchical structuring of behaviour (as reviewed by Botvinick [54]). Hierarchical behaviour concerns the organisation of tasks into a coherent sequence of events, whereas will-nestedness concerns why an agent performs a task in the first place. It should by now be obvious that will-nestedness is more than merely hierarchical structuring of an algorithm, because each ordinal increment of N must involve a higher level goal to provide a ‘purpose’, the fulfilment of which we can interpret as ‘will’.

### 2.3. Constitutive Autonomy and the Creation of ‘Self’

In Farnsworth [14] I argued that we can only attribute agent causation to a system if that system can be identified as a separate agent: specifically, a causal boundary must exist to delineate ‘internal’ from ‘external’, otherwise the term ‘internal control’ has no real meaning. This set the task of finding a structure for which ‘internal’ is causally distinct from ‘external’, giving a clear definition to both. First I note that a parralel approach to mine is presented by Krakauer et al. [55] who seek an empirical means of identifying coherent organisation that suggests life, by using an algorithm which tests hypothetical partitions of system variables in a search for organisational closure and allows for nested “levels of individuality”. Another suggested empirical approach is based on the ‘Markov blanket’—a statistical concept used by Friston [56] and taken up by Kirchhoff et al. [57] with interesting conclusions of relevance to this work, but here I shall take a rout that simply and directly uses causal relationships.

In simple terms, the problem is this: we are used to thinking of individual organisms as causally separate, with clear internal/external separation and conversely thinking of ecological communities as indistinct because they usually have no clear boundaries (see Farnsworth et al. [52]). For a prokaryotic cell, the physical boundary is obviously its tegument, but does this qualify as a causal boundary, given that all living things are materially and thermodynamically open systems? When we extend to a eukaryotic cell that belongs to a multicellular organism, it seems that its boundary cannot provide causal autonomy because each cell must obey the control of the whole organism, which is a community of such cells. The answer is that causal autonomy may be (and most functionally is) partial, so an adequate theory of it must include a means of quantifying its degree (which is what Krakauer et al. [55] provided an empirical method for).

An agent cannot be free unless it is free of exogenous control and this requires that it be autonomous in the sense that its component parts form a system in relational closure [14] (Appendix A). Recall that relational closure means that every part of the system has a causal relation with every other part, so that there is a causal path between any pair of randomly selected component parts. This is a formalisation of the concept of ‘circular causality’ that is described by Rosslenbroich [2]. Relational closure is also the informational structure of the autocatalytic sets conceived by Kauffman [49] and Hordijk and Steel [20] and these in turn embody autopoiesis, from which agent causation might be derived [3]. The relational closure referred to here is specifically *organisational closure*, which is a concept neatly encapsulated in Kauffman [58] term: “Kantian whole”. What is special about such systems is that for any component within them, the state is at least in part a result of only internal as opposed to external causes. This causal structure defines the system boundary in terms of relationships among component parts as it ‘envelopes’ only those parts for which closure is true.

A constitutively autonomous agent is a system for which “internal” is causally distinct from “external”, given a clear definition to both e.g., “every autonomous system is organizationally closed” (Varela [26], p. 58). Most authors conclude that autopoiesis [26,59] fulfils the requirements, but this may be too strong a condition (it is only known to occur in life). Using a mereological argument, I showed [14] that a general agent with the property of transitive causal closure among its parts is a causally autonomous system. Froese et al. [27] distinguished behavioural (based on external behaviour) from constitutive (based on internal organisation) autonomy. This idea has a relatively long history in a multi-disciplinary literature ([27,59,60,61,62] and references therein). The peculiar attribute of a system with constitutive autonomy is that it intrinsically has a ‘self’ to which things may be done and which may do things, including ‘to itself’. This idea would remain esoteric, but for the fact that we can now formally identify and quantify this attribute. Quoting directly from Albantakis and Tononi [42]: “Integrated information theory (IIT) provides a framework for establishing precisely to what extent a system ‘makes a difference’ to itself, from its own intrinsic perspective. The cause-effect structure of a system and its integrated conceptual information Φ characterize what a system ‘is’— how much and in which way it exists for itself, independent of an external observer—rather than what a system happens to be ‘doing’.”

### 2.4. Cognition

As with ‘information’, ‘function’ and other broad terms, cognition has been classically defined in descriptive and non-specific terms that are difficult to operationalise: the ‘text-book definition’ quoted by van Duijn et al. [63] from Neisser [64] is “all the processes by which sensory input is transformed, reduced, elaborated, stored, recovered and used”. The definition of Bourgine and Stewart [65] is more precise: “A system is cognitive if and only if sensory inputs serve to trigger actions in a specific way, so as to satisfy a viability constraint", but is still contested. van Duijn et al. [63] addressed this problem and proposed a definition for minimal cognition based on the two-component signal transduction (TCST) system found in most prokaryotes (using *E. coli* chemotaxis as the example).

Briefly, quoting from both Capra and Laub [66] and Stock et al. [67]: “the TCST consists of a sensor histidine kinase that receives the input stimuli and then phosphorylates a response regulator that effects an appropriate change in cellular physiology. Histidine kinases and response regulators have an intrinsic modularity that separates signal input, phosphotransfer, and output response; this modularity has allowed bacteria to dramatically expand and diversify their signaling capabilities”. The process of perception (through the sensor) has an intrinsically fast dynamic and the response regulation (action) has relatively slow intrinsic dynamic and this difference enables the variety and subtle matching of behaviour to circumstances that amounts to a basic form (perception–action) of cognition. van Duijn et al. [63] specified “embodiment …of a sensorimotor coupling mechanism that subsumes a basic metabolic/autopoietic network” as a requirement for minimal cognition. Significantly, they state that “the sensorimotor organization [of e.g., bacteria] is organizationally autonomous”. By this, they recognised that although it is materially composed of molecular signalling pathways, as an integrated whole, the TCST transcends these: it is a transcendent complex (with the power of downward causation implied by that—see Appendix). For this reason, N≥1, but because bacteria such as *E. coli* do not have any means of altering the inherited information constituting their TCST, other than selecting components of it (e.g., through methylation systems), they are restricted to N=1.

We can think of the TCST as a layer of control and decision making (information structure) within which is nested the basic automaton of a living system (the autopoeitic system and its ‘metabolic reactions’—Moreno et al. [68]). In considering “cell-signalling as a cognitive process”, Koseska and Bastiaens [35] were careful to use the term “reminiscent of cognition”, but the system they focussed on has the characteristic of an emergent level of downward causation, created by the collective behaviour of a network of mutually signalling cells, leading to e.g., the establishment of a differentiated identity for each participating cell, thus forming a tissue and the emergence of tissue-specific cell functions. The perception—action system is made from a molecular interaction network and exerts downward causation by information control [28] upon it, implying that the control system (cybernetic and transcendent) integrates the identity of the tissue or, in the case of the TCST, the behavioural identity of a bacterium. This integration to form the identity (ultimately of an organism) will be a recurrent theme. It arises from the primary integrative role of nervous systems [37] and is necessary for all organisms to gain the condition of ‘wholeness’ before causal autonomy can be achieved [3,13,58].

To generalise, cognition as a multi-layer concept may start (innermost layer) with the basic perception–action process captured by Bourgine and Stewart [65]’s definition. It may then be elaborated (next layer out) to include memory so that different actions can be selected, depending on previous history. A further layer out uses the memory to anticipate environmental states and act accordingly with short term and longer term outcomes being simultaneously represented in some action selection process (see Section 2.5 and Section 2.7). Further elaboration of cognition extends this structure by adding superior layers of goal and action selection and increasing the information content of internal models that represent anticipated outcomes (Section 3.3). In this way, increasingly sophisticated cognition parallels the evolution of information processing structures and conforms to the abstract description of a nested hierarchy: the will-nestedness which I have proposed and for which IIT provides a means of quantifying.

It is informative to note that the simplest realisation of the perceive-act process is a homeostatic loop. This differs from a dynamic equilibrium (e.g., the balance between pressure and gravity that maintains a star) in the following, most fundamental, way. The dynamic attractor of a homeostatic loop is determined by a specific embodied datum, whereas that of the dynamic system is merely a balance of forces. The homeostatic loop consists of a sensory transducer coupled to an internally controlled actuator system, whereas the dynamic system has no actuator and no internal control, hence N=0.

### 2.5. Action Selection

‘Action selection’, defined as the task of resolving conflicts between competing behavioural alternatives [69], is the central example of N>1. To emphasise that action selection is not implemented as literally selecting among *behaviours* (as often assumed), Seth [70] defined behaviour as “observed ongoing agent–environment interactivity”, contrasting that with mechanism: “agent-side structure subserving this interactivity”. Internal decision making is, according to this line of thinking, some process by which actions (that appear to an external observer as behaviours) emerge, not necessarily from explicit arbitration, but generally from internal competition. In the growing literature reporting artificial life simulations designed to elucidate this process, agents share in common an overall objective (usually maintenance of the agent of action by avoiding threats and obtaining required resources). The competition among potential actions is an *implicit* selection guided by an objective function, as opposed to being an explicit ‘executive function’ of some hypothetical higher level module of decision making. This implicit selection either emerges from the interaction among parallel sensor-actuator channels, or is more explicitly expressed in an algorithm in which potential actions are associated with estimates of an objective function (generalised as using the ‘free energy principle’ [71,72]), embedded in a learning process. In the former, optimisation of action selection is performed at an ontological level beyond the individual agent, i.e., emerges through (artificial) selection among competing agents (a simulation of natural selection), often using a genetic algorithm (which was the method used by Seth [70]). In the latter, optimisation of action selection is performed by a learning algorithm that operates at the ontological level of the agent, thus maintaining autonomy. A general account of action selection should be valid for organisms with and without a nervous system, as Baluska and Levin [73] point out, biological systems in general must make decisions about possible activities. A clear and simple example is that of protozoa (e.g., *Stentor coeruleus*) which must decide between feeding and damage evasion behaviours. Although *Stentor* display habituation of response to various stimuli [74,75], in a way that is functionally equivalent to that seen in higher organisms (e.g., the withdrawal response of the marine mollusk *Aplysia* [76]), there is no action selection here. However, these organisms, if sufficiently provoked by repeated noxious stimuli, will detach from their substrate and begin a search for a suitable site of attachment, during which they show no response to stimuli that would normally produce a response (described in Bray [77], quoting Jennings [74]). Such behaviour requires an element of action selection to work. In algorithmic terms it is simple and easily described: two or more ‘subroutines’, each generating a particular perception–action processes, are embedded within a master algorithm with a condition test (an if-then-else clause) responsible for the selection. There is no difficulty in embodying this by living chemistry, since any pathway may be regulated by another molecule acting as a switch and even quite complicated logic circuits can be built (as described in Koseska and Bastiaens [35], Kawano et al. [36], Hagiya et al. [78], Rubens et al. [79]). Thus, even though we do not yet have an explicit molecular account of how protozoa achieve action selection, we should not doubt that mechanisms do exist and can be found.

Prescott [37] emphasised that action selection is part of the wider requirement for behaviour integration, indeed its provision requires the integration of organism actions into a coherent whole. This relates action selection to the essential characteristic of an organism: that it exists as an *organised whole*. We have already seen this idea emerge in relation to cognition and the integration, e.g., of cells into a tissue, or signalling pathways into the coherent behaviour control of a bacterium, thereby creating identity for the tissue or the bacterium, respectively. Vernon et al. [61] expanded the idea that “cognition, perception, and action are all dependent on processes focussed primarily on the maintenance of the agent’s autonomy” to emphasise the role of cognition and action selection in creating *constitutive autonomy* [27] via organisational closure (see Section 2.7). They regard cognition, perception and action as part of a two-way, circular, process: as they say “the reciprocal coupling of action and perception is founded primarily on their roles in the constitutive autonomy of the agent and an associated circular causality of global and local processes of self-regulation, rather than being a mutual sensory-motor contingency that derives from exogenous behavior” [61]. If this is true, then we might interpret the integrating role of any action-perception system as a contribution to an implicit model of the self (Section 3.3), consistent with the idea behind IIT: that the embodiment of intrinsic information by a network creates a self to which effect can be referred [42,80].

### 2.6. Tegument, Transducer and Signal

Despite causal closure, biological systems are both thermodynamically and materially open. They need a flow of energy through them to compensate for energetic gradients (opposing the second law of thermodynamics) and a flow of material for the renewal and growth of their bodies. However, in all living systems, the cybernetic agent is embodied in the fluid phase (i.e., cytoplasm), so a physical boundary is needed to maintain integrity, especially to prevent dilution by dispersion. This obvious practical requirement masks a more fundamental role for the physical boundary of a living system. In the material world, all effects are enacted by physical forces: effect is nothing but the resultant of a set of forces. All forces scale by the number of particles carrying them. All matter responds to the vector sum of forces acting upon it, so chains of cause and effect are never violated. A physical boundary acts as a source of sufficiently strong forces (compared to those likely to impinge from the environment) that the vector sum is dominated by forces created by the boundary, leaving the net effect to be little influenced by its environment.

#### 2.6.1. A Physical Boundary Supports the Cybernetic Boundary

A physical boundary consists of an assembly of molecules, organised so that the forces among them can (up to some limit) greatly exceed those of external influences and also extended over space such that the forces constitute a ‘boundary condition’ for mechanical dynamics. This *organisation of molecules in space* is an embodiment of information and its effect, enacted by the internal forces of the boundary, is one of regulating the magnitude of the exogenous forces that carry dynamic patterns, so that information is retained, but disassociated from the magnitude of its carrier. For example a pressure wave in a fluid (an acoustic pattern) carries information in the variation of pressure that results from oscillatory particle movement (propagated by electrical forces of repulsion among the fluid’s molecules). When it meets a resistant boundary (where inter-molecular forces strongly oppose the force of exogenously applied pressure), particle movement is diminished, the wave is reflected, and on the other side of the boundary it is much diminished. Similarly, as the concentration of a solute in aqueous solution locally increases (e.g., due to a local chemical reaction), thermal (random) forces diffusively disperse it, but when the solute meets a repellant boundary, their momenta are reflected and the concentration remains constant on either side of the boundary.

I conjecture that this effect of a physical boundary is necessary for a cybernetic boundary to exist in practice. If there were no physical boundary between an agent and its surroundings, then any external force would act unaltered throughout the agent and any force initiated by the agent would act equally on the internal and external environment, so no distinction in action could be made between the agent and its surroundings. If no distinction can be made, then no measurement could be made to tell us whether an action arose from within or beyond the agent, so it seems that the agent would not be causally autonomous: condition D1 (there exists a definite entity to which agency may (or may not) be attributed) would not be met. Heylighen [81] points out that in physics and engineering, it is standard practice to consider a dynamic system in isolation, this is implicitly so that it meets the requirements of operational closure (Appendix A), but such isolation is only a tool of thought that requires the application (sometimes implicitly) of artificial boundary conditions. The idea of a living system without an effective and complete physical boundary (e.g., Marshall et al. [46]) is just such an abstraction: in physical reality operational closure requires one.

#### 2.6.2. Transducers Support Agency

An organism must also be able to interact with its environment, if it could not then it would not be meaningfully ‘free’, it would be merely isolated and of course would fail to survive. *Transducers* allow the organism a cybernetic and material connection with its environment without imposing necessary causal determination by the environment. The actions taken by an organism on receiving information and material from the environment are at least partly determined by internal forces that are set in functional arrangements by processes triggered on receiving the output of transducers. In the cybernetic/information domain, this is a case of information controlling information and is only possible because the internal (embodied pattern) information of the organism constrains the range of possible effects that can arise from detecting, e.g., a ligand. It is equivalent to a remote control that switches a lamp on and off—there the control of current in the lamp is only achieved because of specific engineering design and that design is constraining information (this idea is presented more extensively in Farnsworth et al. [13]). In organisms, the embodied information was created by evolution and the information-constrained process is understood as perception and response; taken together as cognition. Cell surface receptors detecting the presence of specific information by ligand binding, then transmit their ‘signal’ to a wide range of intracellular protein networks that are collectively responsible for every aspect of the cell’s life and function. Examples include the chemotaxis system of bacteria such as *E. coli* and the G protein complexes, with associated intracellular transduction (which may include molecular amplification) that lead, for instance to transcription factors regulating gene expression. A more homely example would be the reflex withdrawal of a limb upon painful stimulus. Transducers act as regulators of the magnitude of causal force, allowing the information that forces might carry to pass through, but disassociating that from the magnitude of these forces.

In general, transducers are the means by which cause and effect is transformed into signal and response, but only in the context of a barrier to the force that is being exerted. A transducer is the material embodiment of information that specifies a communications channel. It is an embodied pattern which allows information to pass from its external side to its internal (or vice versa) and in the context of a boundary it determines the number of molecules that carry the information. By disassociating the information from the force which carries it, the essential effect of transducers is to transform causes into signals so that their effects can be rendered as responses (which thereby may become optional). This definition of transducer includes the obvious cell-surface receptor molecules, but extends to most trans-membrane protein structures for which a function is known, especially those forming selective channels (both active and passive transporters). In many (e.g., ligand gated channels), the sensitivity of the transducer is controllable (emphasising the optionality of response). It may seem wrong to include e.g., ion channels as transducers, but from an information/cybernetic perspective, molecules are (functionally) patterns with electrostatic surface variation that carries a ‘signal’. This signal is the information and the amplitude (regulated by the transducer) is the number of (signal repeating) molecules that impinge on the cell surface and may be permitted through.

### 2.7. Signals and Nerves As Integrating Mechanisms

Nerves provide a universal interface between any kind of receptor and any other (but especially motor) system in the body of an animal: they are transducers for multicellular animals that operate at the supra-cellular level. Nervous systems perform two categories of function that were termed input-output (act-state) and internal (act-act) coordination by Jékely et al. [82], the former being the selection and implementation of appropriate actions for a given state of the environment and the latter being the coordination of micro-actions into a coherent whole. Having suggested that in organisms, a ‘self’ identity is created by integration of information flows in its highest level of organisation (recognising that the levels are mutually dependent in real systems) and that this is manifested as operational closure, I emphasise the internal coordination function here.

New insights into the evolution of neurons are still emerging [83], with recent understanding of the relationship between their evolving function and physiological mechanisms being relevant to the development of their increasing integrative power. Brunet and Arendt [84] proposed an explanation for the evolution of nerves from early eukaryotes, initially as a tegument damage repair response to calcium ion influx (involving cortical actomyosin and mechanosensitive Ca${}^{2+}$ channels), developing to an escape response, demonstrated by the amoeboid movement mechanism and the control of flagellar beating and integration of cilary beating (elaborated by Jékely [85]) which led to the development of action potentials via voltage-sensitive ion channels and the eventual spread of these mechanisms to whole cells. This enabled the development of mechanosensory contractile cells from which neurons and myocytes emerged by devision of labour. Brunet and Arendt [84] say “Following this scenario, various sensory, secretary and contractile modules and functions were segregated into different cell-types, so that the depolarization-secretion and depolarization-contraction couplings became the functional core of neuron and myocyte physiology respectively”. The action potential system is essential for long-range communication via axons, but additionally the ability of neurons to generate action potentials upon ligand binding and to secrete the relevant ligands has enabled the growth of arbitrarily complex networks of cybernetic control supported by integrative ganglia and further by brains.

The effect has been a considerable expansion of the cybernetic system underlying cognition and forming that integrative transcendent complex which we observed to be most primitively embodied in the two-component signal transduction system of bacteria. The integrating role of nervous systems is not restricted to behaviour generation, but includes the integration of physiology and development (especially growth, metamorphosis, and regeneration) via hormone signals produced by neurons [82] and we can consider integration of physiology and development to be constitutive roles. Vernon et al. [61] used the distinction between constitutive and behavioural autonomy proposed by Froese et al. [27] to clarify that integration by cognition serves both the maintenance of constitutive integrity (organisational closure) and the generation of behaviours that manifest at the (organisational) level of the individual organism. They start with homeostasis in a perturbing environment, whereby the organism may deploy a “hierarchy of homeostatic self-regulatory processes” that extend from ‘reflexes’ through motivational ‘drives’ to “emotions and feelings, often linked to higher cognitive functions” to counter threats to the organism’s autonomy (as they put it). This hierarchy reflects increasing constitutive organisation, which they represent on the two (conceptually) orthogonal axes of behavioural and internal organisation, so that collectively constitutive organisation emerges from correlated increases in both axes. At each rise in the level of constitutive organisation, new integrative functions (of nervous systems) are recruited. Vernon et al. [61] further propose ‘allostatic’ regulation (distinct from homeostasis because it anticipates perturbation) as the process behind motivational drives, whereby associative learning allows prediction and the sequencing of behaviours, coinciding with the emergence of ‘somatic modulation’. This, they argue, is then the foundation for building multi-sequenced behaviours that are informed by interoception and internal stimulation of behaviour prior to action (all this is summarised in Figure 4 of Vernon et al. [61]).

#### Memory: Why It Is Needed and How It Is Implemented in Biological Systems

Anticipation requires stored information (memory) and for simple organisms, the only option for this may be inheriting information from the parent(s), in which case N≤1. A finite automaton may respond to changes in its environment that are predictable over evolutionary time-scales (e.g., diurnal, tidal or sporadic, such as rainfall), using information that is incorporated into its cybernetic design by natural selection, i.e., *given* information. This is strictly limited by the information capacity of the organism design. To extend anticipation beyond this limit, the organism must incorporate *mutual information* (a generalisation of correlation) with environmental variables, the mutual information being instantiated within its *informationally-closed* system [55]. This information is a time dependent (dynamic) addition to the organism; not only that, but the mutual information must also be functional in that it must inform action selection. The most sophisticated level of cognition recognised by Vernon et al. [61] involved learning, which specifically requires the organism to create its own information representing the relevant behaviour of its environment (or itself) and which permits the creation, or alteration, of motivational *goals*, including through interoception (see Seth [86] for an expanded account).

Incorporated information that is mutual with the sensed environment is an example of embodied information that is created by an organism through the action of already embodied information. So any organism with a *dynamic* memory is in principle capable of supporting N>1. This is one interpretation of why memory is one of the foundations of autonomous will, but its existence is neither a sufficient nor a necessary condition. There are many examples where a crude form of memory is present and necessary for the operation of an organism, where N=1 and we still refer to these organisms as autonomous agents, (for example bacteria that inherit all their embodied information are still considered autonomous). The critical difference between these and organisms that can learn, is that in learning the dynamic mutual information is incorporated into the cybernetic structure of the organism, whereas the memory of e.g., the gradient sensing mechanism of chemotaxis is not (it is therefore ephemeral). The ability of neurons to form networks via synapses and change their sensitivity, both as transmitters and receivers of neurotransmitters, has enabled a revolution in the ability of organisms to store their own information. A great deal is now known about the mechanisms involved, though uncertainty remains over many components (see Titley et al. [87] and Sweatt [88] for recent reviews). Neural plasticity and the adaptive growth of neurons and their synapses gives neural networks the ability to form potentially any cybernetic system and they have long been the inspiration behind cybernetic models of control systems in general (e.g., Ashby [89]).

## 3. The Evolution of Self-Control

As self-sustaining dynamic systems, stars have ‘thermodynamic autonomy’, supported by negative feedback, but they cannot evolve. That is not because they are free from natural selection, but because they cannot inherit the traits of their parents; i.e., there is no information storage and therefore no inter-generation information transfer. This is a reminder that at the cybernetic level, evolution by natural selection is fundamentally a process of filtration of (random) data (Appendix A) to generate information in the communication of data from one generation to the next. This process is essential for the creation and maintenance of information that represents a *goal* in any organism. Living organisms seem to uniquely qualify for terms such as goal, purpose and will, but the apparently normative connotations of these terms lead to their rejection by many, especially strictly empirical, scientists. In more precise and scientific language, we can now say that living systems demonstrate the characteristic of causal autonomy, since causal autonomy implies the presence of a goal, located in an mapping between perception and action that follows an objective function, such as the ‘free energy principle’ of Friston [71]. One unifying characteristic of living organisms, then, is their embodying of a goal and objective function as information that is inherited and is (continually and dynamically) optimised for reproductive success by the process of natural selection. In short, we can say that for all organisms N≥1.

### 3.1. Re-Programmable Organisms: Digital and Analogue

If a system is to have N>1 (meaning that it has the ability to determine its own responses), then it needs a way to change the cybernetic control of its actions (i.e., its internal informational structure) and one obvious way to do that is by reprogramming. Walker and Davies [31] focus on computation in explaining the origin of life, referring to genetics-first theories as ‘digital-first’, emphasising the need for ‘programmability’ and its provision by informational polymers (the genetic oligomers RNA, DNA etc., plus peptides), but I will argue that all aspects of organisms are both digital and analogue. The substantial and varied two-way interactions between genetic information and that carried by cell signalling pathways shows that reprogramming at the cellular level is usually effected by variation of the *expression* of genes: its selection and timing, which in turn is under the ’master control’ of a genetically determined algorithm. Organisms with a nervous system can potentially change their responses, because the nervous system is able to store (and recall) relevant information. In this context, therefore, we are concerned with *re-programmable* systems more than merely programmable.

A re-programmable system is a cybernetic system which can generate a different outcome for a given set of stimuli, depending on the information embodied, which may be changed by internal or external means. Re-programmability is the property of a system that allows its state to change reversibly, approximately independent of energy flow: i.e., changes of state are not accompanied by substantial changes in potential (embodied) energy [14]. If significant changes in energy were associated with cybernetic changes then those changes would always be biased by the difference in energetic cost between one information pattern and another. Since the elementary unit of information control is the switch, energetically unbiased switching is the physical underlying mechanism of ‘information abstraction’ referred to by Walker and Davies [31]. The information polymers share a unique and crucial property: in terms of polymerisation, their monomers are chemically equivalent, but because they include different molecular units as ‘side branches’, they present functional differences that can be ‘recognised by’ (i.e., correlated with) other molecules. All nucleotides are joined by phosphodiester bonds, irrespective of their identity. Amino acids likewise, all having an amino group at one end and a carboxyl group at the other, join in any order with very nearly the same change in Gibbs free energy. In both cases, therefore, the order of monomers in an information oligomer chain is thermodynamically indifferent: every sequence of the same length and monomer composition is created with essentially the same Gibbs free energy. This is the chemical basis of programability: processes such as site-specific recombination and transposition enact re-programming at the genetic level (e.g., in vertebrate immune systems), but this is only possible because the monomers involved, like a row of switches, can be rearranged in any order to instantiate a different pattern: a different message.

In communications/information theory, analogue is distinct from digital because it is continuous, rather than discrete. At the molecular level, biological systems are inherently digital (in the communications theory sense) since they consist of interactions among individual molecules that either bind, react, cleave, or change conformation, or they do not. Although convenient for description, systems of interactions among individual molecules are not usually functional; it is at the scale of populations of molecules (of the order $>10^{5}$, as Luisi [90] reminds us) that we see function emerge and because natural random variations among the interactions disperse their action in time and space, the aggregate (course-grained) result is effectively analogue (just as it is in the quintessential case of gas mechanics). When a system is composed of multiple interacting reactions then the system may be *represented* by a discrete algorithmic (cybernetic) approximation, as in the case of the *E. coli* chemotaxis system.

The digital/analogue distinction is much more profound in cybernetic theory [11], where digital is defined as symbolic representation whereas analogue describes the situation where the physical structure *is* the information (the structure embodies and is defined by the information—see discussion in Farnsworth et al. [12]). In this case, the information represented by a cybernetic system is only an interpretation (a model) of the physical phenomenon that exists (e.g., an analogue pressure gauge, represented by the information it indicates). In the analogue system, there is a physical relation between the informative phenomenon and its cause. In the digital, there is not: for example, a particular pressure may be represented by any particular—arbitrarily chosen—configuration of e.g., lights on and off. The digital representation of information and its recognition are *designed* for the purpose of communication, whereas the analogue information is intrinsic to the physical system embodying it and is causally linked to its surroundings by physical forces. Biological chemistry is analogue in this sense. Even information polymers embody and convey information by analogue means in this cybernetic theory sense. Since most cellular chemistry and certainly most DNA/RNA information is discrete, it is digital in the information theory sense and analogue in the cybernetic theory sense.

As well as informational polymers, biochemical signalling systems can be reprogrammed (as in *Stentor* habituation). Kunita et al. [91] showed that protozoa can ‘remember’ the boundaries of a confined swimming space. The mechanism they proposed is, at the cybernetic level, an elaboration of the chemotaxis controller, with the important addition of a strain receptor (Ca${}^{2+}$ channel) located at the anterior of the *Paramecium* they studied. This is especially interesting in the context of the evolution of nervous control by adaptation of such channels in conjunction with Na${}^{+}$ and K${}^{+}$ to form depolarisation activated channels from which action potentials and the functions of neurons might have developed [84].

At the higher level of neurons, the biological basis of learning and memory, is itself constructed from biochemical signalling systems: those underlying long-term potentiation (LTP) and long-term depression (LTD) of synaptic connections (see Shimizu et al. [92]). For example, the development of neural networks by neuronal cell proliferation, axon elongation and synaptic plasticity (LTP, LTD) is regulated by (among others) the multi-purpose cell-signalling glycoprotein ‘Sonic Hedgehog’ [93]. This, in turn, can reprogram cells at the genetic level, for example the expression of the ‘early growth response’ gene *Egr-1* in learning [94] and emotion [95]. The classical conditioning of withdrawal response in the marine mollusk *Aplysia* is now the text-book example of neural reprogramming mediated by a simple form of LTP [76]. Operant conditioning, in which an organism learns to respond to a reward by increasing a behaviour with which it correlates, has also been demonstrated at behavioural, neural and molecular levels of feeding control (buccal motor programmes) in *Aplysia*, where dopamine provides a ‘reward signal’ [96].

This reward signal is very significant, because it carries the information about the distance between the current state of the organism and its optimal state (hence it acts as an objective function for a goal). It is a local (and analogue) representation of the fitness gradient currently experienced by the organism in its present environment and is constructed internally by processing the information received from the organism’s receptors (i.e., by perception). Chemical pathways are so arranged by inclusion of feed-back and feed-forward loops, etc. that the reward signal is (automatically) maximised by adjusting the organism’s actions. This is exactly what we see in chemotaxis, but operant conditioning shows it in action at a higher level of generalisation: one in which the chemical pathways themselves are plastic and under the control of a higher level algorithm. We could liken this to some hypothetical (not known to exist) bacterium in which the parameters of the chemotaxis controller were themselves under internal control. This arrangement constitutes a two-level hierarchical algorithm in which (crucially) the higher level is goal-seeking and enacts that behaviour by manipulating the lower level control and all this is achieved in analogue computation. It may be speculated that this sort of two-level biochemical control algorithm lies behind the behavioural repertoire of *Stentor*.

### 3.2. Goal, Purpose and Will

The maximisation of a reward signal is the simplest form of goal seeking behaviour, since it does not *explicitly* specify a goal, only the direction towards it (as in the free-energy principle [71]). A goal is a specific piece of information embodied in a system. Taking a familiar non-biological example, the set point may be *programmed* into a digital thermostatic controller, or be a parameter of the design of an analogue one. For organisms, goals are ultimately an expression of evolution by natural selection: the top level goal for all organisms is the inherited drive for fitness (lifetime reproduction success), which we have seen may be internally represented by a reward signal. Since fitness maximisation is an objective with a purpose, its goal is referred to as a ‘purpose goal’. For simple organisms this top-level goal is the only purpose goal present, which is why N=1 for them. Even for them, though, fitness maximisation implies a *purpose*, though it is simple and not the ‘choice’ of the organism.

The meaning of purpose here is not normative, it only specifies the existence of a goal and goal seeking behaviour, but since we have established the organism as an integrated whole with causal closure, it is specifically attributed to the organism. This purpose is a common feature of all organisms and could be considered one of the defining features of life (after all, every living cell today, whether ‘free living’ or part of a multicellular organism, has been produced by fission of a parent cell, forming a unifying chain that could in principle be traced back to the first common ancestor of all extant cells). Crucially, the top-level goal is maintained at the level of integrated organism, not the individual cell (where it would generate a cancer). This means that the top-level goal of fitness maximisation is to be found in the transcendent complex that integrates the organism as a whole and nowhere else. It is largely for this reason that I propose that as the will-nestedness of organisms increases with their behavioural complexity, it is by devolution (creating another lower level) rather than emergence (creating another higher level).

In Farnsworth [14], I expressed goal and purpose in mathematical notation, relating them to ‘function’. Since the goal is a fixed point in an objective function, it constitutes information (e.g., a homeostatic set-point) that must be embodied in the agent’s internal organisation. At the top level, the objective function represents the overall functioning of the organism. A precise definition of ‘function’ is important here as it emphasises the relationship between a component (or sub-system) and the organism of which it is a part. ‘A function is a process enacted by a system A at organisational level *L* which influences one or more processes of a system B at level L+1, of which A is a component part”—Farnsworth et al. [52], based on Cummins [97]. This implies that the function of any part of an organism is predicated on the function of the whole, represented by the objective function. This idea was previously expressed by Jaeger and Calkins [21] who termed the highest level function, from which we identify the *purpose*, as the ‘master function’. From this and an argument based on causation, I derived the meaning of ‘will’ (cause attributable to an agent) to be the source of what the organism strives towards, so the ‘will’ of an organism was identified as the goal of the objective function. This goal is information instantiated in the form (pattern) of the organism-level transcendent complex (a detailed argument for these deductions was presented in Farnsworth [14]). This means that the organism (as the embodiment of the top-level transcendent complex) and its ‘will’ are not separate, but mutually forming.

These ideas parallel the analysis of Ellis [18] and Butterfield [98] who resolved downward causation into a mathematical description of control structures. The second (of five) categories of downward causation identified by Ellis [18] was ‘non-adaptive information control’ in which “higher level entities influence lower level entities so as to attain specific fixed goals through the existence of feedback control loops”. The third type: downward causation “via adaptive selection” refers to fitness criteria as “meta-goals” and it is clear that these originate before and beyond the existence of the organism. Ellis [18] makes it clear that the meta-goal is “the higher level ‘purpose’ that guides the dynamics” explaining that “the goals are established through the process of natural selection”. This was amplified by Walker [15] who stated that downward causation must always be directed by a purpose, for which we need to identify a goal.

Generalising only slightly, I showed [14] that we can envisage a nested hierarchy of goal-driven systems and for each, the ‘goal’ is the source of *causal power* and as such may be identified as the ‘will’ (free or otherwise). An organism with at least two nested levels of causal power, the higher of which, at least, is embodied *within* it, has a ‘will’ to determine its ‘will’, which conforms to the philosophical notion of *will-setting*. This concept has been formalised by the discrete variable ‘will-nestedness’ N introduced previously. The philosophy of free-will recognises that ‘will-setting’ is needed in addition to unconstrained will-enactment for ‘free will’ to exist [99,100,101,102] and its appearance seems to be unique to life [14].

### 3.3. Implementing Higher Will-Nestedness with Brains

In more complex organisms, the development of memory has enabled a model of the self, by which goals can be modified and new goals created internally in accordance with the top-level goal of life’s ‘master-function’ (enabling N≥2). Lower level goals are constructed by internal modelling as increasingly complex tasks are compartmentalised. The master function specifies the criteria needed to assess possible future reactions to the environment. Organisms with a central nervous system (and emotions) can implement more complex (information rich) and adaptable (internally branched) algorithms for the master function, and these are instantiated hierarchically (at least in cybernetic terms). Assessing possible future reactions before they are enacted requires the agent to maintain an internal representation of itself, where hypotheses about the outcome can be tested before commitment. Hierarchical reinforcement learning has become the standard model for this process [54,103,104,105]. From a different perspective, at least a closely related process was represented by the hypothetical ‘free-will machine’ presented in Farnsworth [14], which is nothing more than a cybernetic construct: a circuit of computational elements interfacing with the environment via transducers and actuators. It consists of two Turing machine computers, one maintaining a model of the self and the other estimating possible futures given candidate behaviours, which are then assessed against goals using a finite state automaton computer, which selects and passes onto actuator machinery the chosen action, which when acted, leads to environmental feedback that informs the model of self (via transducers) to complete an adaptive cycle. Turing machines are readily created by neural networks, indeed neural networks, even without an explicit memory, are known to be able to implement any Turing machine [106] and working memory is created by the action of neural networks (e.g., in insects), so a cascade of Turing machines is a plausible model.

I propose that the free will machine is analogous to the basal ganglia (BG) of vertebrate brains or the central complex (CX) of insect brains (compared by Fiore et al. [107]). When these neuroanatomical structures are removed or disabled in living vertebrates or insects respectively, the result is an animal that can walk zombie-like without being able to modify its walking in response to stimuli. In conjunction with other (specialist) circuits, the BG and CX have the power of action selection [107,108], taking account of many different stimuli simultaneously and show the properties described by Redgrave et al. [109]: they have inputs carrying information about both internal and external cues relevant to decision making; they can ‘calculate’ the salience, i.e., the urgency that should be accorded each available action; there is a mechanism for resolving conflicts among competing actions according to their saliency and the outputs allow the ‘winning’ actions through, whilst blocking the losers [108]. The measure of salience is self-evidently consistent with the fitness imperative which I nominated as the organism’s master function. The BG and CX structures seem therefore to already be providing the organism with N≥2, as my ‘free will machine’ [14] was designed to illustrate.

I offer the following conjecture: that as the number and range of both information inputs and possible actions of an organism has increased through evolutionary time, the hierarchical nesting of goal-directed systems has increased *from the top-down*. The increasing scope of the information processing task (connecting sensory input to behavioural output) has led to, and been supported by, an increased mass of neural tissue in more differentiated structures. Lower-level control has gradually been left as the task of more ancient brain regions, whilst top-level control has migrated, e.g., into the neocortex among mammals. A hint of this is provided by development, where for example, the optokinetic nystagmus (an image-eye movement coordination reflex) of primates resides in pre-cortical regions in neonates before being taken over by the cerebrum in adults [110].

### 3.4. On Consciousness and ‘Free Will’

Some philosophers object to the attribution of freedom of decision-making to structures such as the CX and BG and to terming my model of autonomous decision making as a ‘free will machine’. For them free will is something only attributable to a conscious mind, separate from the ‘zombie’ body and certainly not attributable to a neural structure or, perhaps worst of all, a human artefact (a robot). My position on this is that the objection depends on what we identify as the entity exercising free will. The objectors are right to point out that, for example an algorithm incorporating a model of the self and decision rules, is not what we would normally think of as having moral responsibility for its actions, but that is because we have inherited a culture in which the idea of free will is inseparable from moral responsibility and which assumes that moral responsibility can only be attributed to a conscious human mind [100]. Philosophers following this tradition position free will with the conscious mind, which we would then have to locate, either in the body, or somewhere else. One of my propositions is that the functional identity of an organism is the top-level integrating information, existing as a transcendent complex which exercises downward causation over its parts, ultimately obeying the biological master function (but recognising that causation is both upward, same level and downward and the levels are not separable in real systems). If this is true of all organisms, then it is true for a human being, hence their free will is that of the particular instantiation of the informational structure that is them. Its physical manifestation is implemented by embodiment in the same sort of ‘wetware’ as is found in the bacterial chemotaxis controller and the neural networks of nematodes and sea-slugs and it includes the basal ganglia as well as a cerebrum.

Brain pathologies have clarified that the high-level behaviours we normally think of as giving a person their free will are often (but not exclusively) associated with the cerebral cortex and the same is true of what we normally think of as ‘consciousness’. It seems likely, though, that through the evolution of the brain (see Smart [111]), which has responded to increasingly rich input information and an expanding option-space, the executive functions that manifest as conscious thought have migrated into the neocortex as it expanded, but that the top-level control (the transcendent complex that is the identity of the organism) remains *multiply-realisable* and specifically not trapped in any particular anatomical region, indeed if my proposition is correct, it is necessarily distributed so as to integrate the organism as a whole: it is an example of ‘embodied consciousness’ [61,86,112]. If one accepts determinism (the materialist stance that there is, at any instant, exactly one physically possible future [113,114] (rather than absence of randomness)), the question of free will for any organism can only apply to the cybernetic control that integrates the organism and, by its organisational (transitive) closure, gives the organism a definite (separate) informational identity, whilst its material parts are all continuous with the rest of its environment (it is materially and thermodynamically open). In this sense, the question of free will is one of identity: it should address an information structure having causal power (in principle quantifiable by IIT) and which most precisely describes the organism. This is illustrated by the observation that organisms of many (possibly all) kinds show consistent individual-attributed differences in behaviour, often termed ‘temperament’ or, less carefully ‘personalty’ (especially studied in terms of the ‘boldness axis’). What is responsible for their differences? We could hypothetically trace back through their life’s experiences and their inherited lineage, including all the influences that are part of a chain of cause and effect that lead back to a time before life on earth. Alternatively we could (more practically) attribute the differences to the organisms themselves, not the material composition, but the informational structure which makes them an integrated and informational whole, different from any other.

The idea that consciousness is not located in a particular structure or even a particular part or process of the brain, but rather is distributed and emergent from the integrated whole, is consistent with the ideas of Tononi [6] and colleagues and also consistent with the distributed concept of consciousness proposed by the philosopher Daniel Dennett (e.g., [115]). IIT theory implies that consciousness is not an all-or-nothing property, but rather is a variable which can be quantified. Since the ‘free will machine’ and models of the BG and CX must embody integrated information, IIT would (controversially) attribute at least some degree of consciousness to these structures. Furthermore, these structures only function in conjunction with the rest of the central nervous system, the peripheral nervous system and, crucially, all the living parts of the body, i.e., in the context of an integrated whole, again suggesting embodied consciousness. It is this whole that I propose to identify as the organism and I would agree with those who argue that consciousness cannot be identified separately from it.

## 4. Conclusions—The Evolution of Causal Independence Through Information Acquisition

I have argued that agency is a (perhaps the defining) special feature of organisms and that it is enabled by the combination of autonomy and purpose, the latter being defined by a goal in an objective function. Autonomy results from organisational closure which is ensured by autopoiesis (itself, a special case of organisational closure). The goal-seeking emerges from evolution by natural selection: only those autonomous systems having a fitness-maximising objective function can compete for resources and thereby persist in the biosphere. Organisms are not so much the material entities that we observe, they are more profoundly the information structures that give integrated identity to a system of causal relationships: they are essentially cybernetic phenomena. For the simplest organisms, this cybernetic construct can be modelled as a Finite State Automaton with a master (objective) function and both the FSA algorithm and the objective function (jointly necessary for life) exist as embodied information that was inherited from a parent and slowly refined by filtering random data through natural selection. This has been possible because organisms communicate (across generations) information in the form of re-programmable, but stable data, embodied in information polymers. The constitutive autonomy of autopoiesis is made possible by maintaining unbroken the tegument that has enveloped living cytoplasm since the first common ancestor of all extant organisms.

As organism designs proliferated, ecological niches emerged and expanded, enriching the potential option space for organisms and thereby creating opportunity for the branching of the master function into sub-routine automata with associated, subordinate (and more specialised) goals. This led to the reforming of the top-level control into a process of action selection. This was embodied, first by more complicated (information rich) intracellular signalling circuits and then, developing from one or more of these, alongside multicellularity with cell differentiation, it led to mechanosensory contractile cells which differentiated into to neurons and myocytes, taking the specialised roles of perception and action: the first cognitive systems emerged. Nervous systems then enabled the formation of working and long term memory and the creation of internal models of the self in the context of the environment, enabling action selection to gain further layers of context-specific goal-seeking structure: the appearance of ‘executive function’. The top-level information control was preserved throughout this evolutionary development: in the further elaboration of nervous systems, including the emergence of the neocortex and the migration of executive function into this computationally powerful anatomical structure. At every stage, it is the top-level transcendent complex (i.e., the information structure with causal power over its parts) that integrates the organism into a whole and which, by its embedded master function, defines the ‘will’ of the organism. The evolution of apparently increasing powers of decision making has been a top-down process whereby the highest level has devolved control to an increasing number of nested layers of subordinate controls, despite the anatomical appearance of increasingly powerful executive functions emerging from apparently less autonomous structures. To examine these ideas more precisely, I have proposed will-nestedness as a representation of the number of nested layers of goal-directed control that are embodied within an organism. Will-nestedness suggests the following stages in the evolution of organism autonomy:

- perception and homeostasis: 1 goal N=1,
- action selection: 2 goals (N=2),
- learning, leading to an adaptive self-model and extension beyond 2 goals (executive function): N≥2).

The recent application of IIT [45,46] has shown that the nested hierarchy of information control that constitutes the causal independence of organisms is susceptible to quantitative scientific enquiry and I hope that it will be stimulated by my synthesising perspective. To that end, I offer the following proposition to examine, discuss and test:

*that organism identity is created by the information structure, (particularly including that at the highest level of causal closure at which the highest level of will-nestedness is identified) and that this coincides with the ‘maximally irreducible cause-effect structure’ defined in IIT* [7].

## Appendix A. Definitions

**Ensemble**: any set of physical entities, e.g., molecules, arranged in time and space. The ensemble is the basis for information to be embodied because information can only exist as a configuration of energy/matter in time and space (compare with Landauer’s dictum) (see Farnsworth et al. [12]).

**Data**: a *particular* configuration (arrangement) of an ensemble. This is termed a micro-state in physics, a sequence or simply data in computer science and bioinformatics. Data is not about anything, it is literally just a configuration: the word refers to an entity, not the properties of an entity (see Floridi [116]). Data, in this sense, is called ‘intrinsic information’ in IIT, which was developed in terms of “information generated” and “specified” by systems of related sub-systems: e.g., Tononi and Koch [80] write: “Information refers to how a system …specifies a form (‘informs’ a conceptual structure) in the space of possibilities”. For example, the specific order of (an assembly of) amino acids in a peptide (ensemble) is data which specifies the form of a protein molecule.

**Intropy**: is a short form of the term ‘information entropy’, stating the number of ways an ensemble can be arranged. This is the capacity of the ensemble to hold data because each unique arrangement of the ensemble is a unique datum. Intropy is a statistical property of the ensemble, it is a measure of an ensemble, rather than the ensemble itself. It is defined by the same equation as that defining Shannon’s ‘entropy’ (see Adami [117] for an introduction to this and related concepts).

**Pattern**: any configuration (data) embodied by an ensemble which has non-zero auto-mutual information at any trans-location distance (delay or shift when referring to a sequence). Pattern therefore refers to a special kind of data, not the properties of that data. Pattern is the special case of data with the property that if the data were bisected at a particular point, one part would hold relational information (see below) about the other. The specific configuration of atoms to form a molecule is an example of a pattern.

**Pattern information**: the quantification of a pattern in terms of information properties, especially its maximally compressed size in bits (calculated by e.g., its Kolmogorov complexity). In this sense information is to pattern (or more generally data) as mass is to matter (see Farnsworth et al. [12]).

**Relational information**: the mutual information (*sensu* statistics) among data. This is what Shannon referred to as ‘information’. Every datum is a potential source for relational information, so its properties (e.g., pattern information) can be calculated, but this is not yet realised as Shannon’s information until it is partnered with (related to) another datum. Relational information is strictly a relational phenomenon that cannot be attributed to any single datum or pattern. In general it is a property of an ensemble of data. Since it is a measure of mutual information, it is a metric describing entities, not the entities themselves. Adami [117] explains relational information (though he does not use that term) and mutual information in relation to information biopolymers (e.g., RNA, DNA and proteins).

**Effective cause**: is a physical effect of one agent upon another (e.g., moving it, or transforming it in some way, or preventing one of these). Effective cause is always produced by the action of physical forces on matter/energy.

**Causal power** (conversely) is attributed to an agency which can influence a system to change outcomes, but does not necessarily itself bring about a physical change by direct interaction with another agent. We may attribute information with causal power only if it can be shown to influence the outcome of an interaction in the physical world (i.e., via effective cause). An obvious biological example is the formation of proteins in the ribosome, based on RNA pattern; a more subtle one is to be found in the ‘Hedgehog’ signalling pathway (see Yao et al. [93]) and one clouded by multiple layers of complexity is the ‘landscape of fear’ which influences the behaviour of prey organisms [118]. In all cases, pattern determines boundary conditions constraining the effect of forces so that outcomes (effects) carry ‘mutual information’ (a generalisation of correlation) with the patterns that constrained them. The concept of effective cause should be compared to ‘cause-effect repertoire’ in Oizumi et al. [7].

**Effective information**: any pattern (measured by pattern information) with causal power over any pattern when it interacts with another pattern to form relational information (the change may be in the partner pattern, itself or any other). Effective information measures the mutual information between an agent with causal power and the pattern it effects, which may arise alongside the formation of relational information (see Walker and Davies [31]). Effective information can only be observed when patterns form relational information, but is not itself a property of relational information. The reason is that a pattern may cause an effect in non-pattern to form a pattern (e.g., the RNA-ribosome complex makes patterns from an unordered mix of amino acid substrates), or it may cause a repeatable effect in relation to a range of partner patterns (e.g., the action of a cleaving enzyme). It is still context dependent though, since its actions depend on what, if anything, it interacts with. Note that in general change is not necessary: the effect may be to maintain stasis (i.e., to inhibit change). In this sense, a pattern may be effective on itself because it maintains its form (e.g., in a crystal the ensemble pattern is self-reinforcing with the effect of inhibiting dissolution). Effective information should be compared to the definition of ‘cause / effect information’ in Oizumi et al. [7].

**Transcendent complex**: A transcendent complex is an informational structure emerging from an interacting set of components and specifically refers to the information that is embodied in the interaction network, separately and above that embodied in the component parts. As such, it may be quantified using the IIT measure of integrated information φ. Interacting patterns can form ‘transcendent complexes’ [52] that have actions which can be attributed to the organisational level of the complex—this being a higher organisational level than that of the patterns composing it. Specifically a transcendent complex is the pattern existing at the higher organisational level over and above that of the sum of its component parts (it is termed ‘transcendent’ because it is independent of the way it is embodied—it is multiply realisable). In turn the complex may interact with other complexes to result in actions at a higher level still. The hierarchical nesting of such relationships is the structure of life [12]. It should be noted that φ refers to effective information, since it measures the proportion of total behaviour that is caused by the structure under observation. For a transcendent complex to be effective, it must include effective information and φ can be used to quantify this.

**Functional information**: when the effect of effective information operates at a higher organisational level, then its effect can be called functional [52]. Note this does not imply a purpose (as many attempts to define function have). For example, a mutant gene has an effect when it is expressed and this effect can be described as a change in cell functioning (i.e., at a higher organisational level compared to gene expression): thus the gene (pattern) is functional at the higher level of the cell (see Farnsworth et al. [52]).We can quantify functional information through estimating the effective information which is found at the system level (i.e., the level of the transcendent complex). Functional information should be compared to the definition of ‘concept’ in Oizumi et al. [7] and its metric: integrated information.

**Closure**: is a mathematical concept applying to sets of relations. In general, a set ‘has closure under an operation’ if performance of that operation on members of the set always produces a member of that same set: this is the general definition of *operational closure*. More precisely: if *C* is a closure on set *A*, it means $C:A\to A^{\prime }$, *iff* $A\subseteq A^{\prime }\wedge {\left(A^{\prime }\right)}^{\prime }=A^{\prime }\wedge A\subset B\Rightarrow A^{\prime }\subset B^{\prime }$. It can be applied in many relevant systems, for example if X is a set of chemical species and R a set of reactions, then if for every possible reaction in R among members of X, the products are always also members of X, then the set X is closed under R (this is one of the prerequisites of autocatalytic sets). The idea of closure is important in cybernetics, where organisational and operational closure are distinguished. According to Heylighen [81], “In cybernetics, a system is *organisationally closed* if its internal processes produce its own organisation” (my emphasis). This was used in the ‘Closure Thesis’ underpinning the theory of autopoiesis: every autonomous system is operationally closed. Quoting Varela [26] “A domain K has closure if all the operations defined in it remain with the same domain. The operation of the system has therefore closure, if the results of its action remain within the system itself”. More loosely, Vernon et al. [61] stated that “the term operational closure is appropriate when one wants to identify any system that is identified by an observer to be self-contained and parametrically coupled with its environment but not controlled by the environment. On the other hand, organizational closure characterizes an operationally-closed system that exhibits some form of self-production or self-construction” [119]. Rosen [60] used the Aristotelean language to describe the special case of *causal closure*, calling it “closure to efficient cause”. This is an operational closure in which the operation is causal. This idea was formalised by a mereological argument in Farnsworth [14] to define systems with an inherent cybernetic boundary (where inside is definitively separate from outside) as only those systems having transitive closure for causation: (xCy: read as *x* causes *y*), meaning that the state of an object *y* is strictly determined by the state of the object *x*. The *transitive causal closure* of a system means that its components form a set A of causally related objects under *C* such that there is no object in A who’s state is not caused by an object in A: every part of the system is causally connected to every other. It is from such systems that a transcendent complex may arise. Finally, taking causation as manifest in *mutual information*, Bertschinger et al. [120] derived a quantitative metric of *information closure* to operationalise these concepts in systems theory, which has developed into an information-theoretic method for system identification [121,122].

**Autonomy**: I take the useful definition provided by Vernon et al. [61] “the degree of self-determination of a system, i.e., the degree to which a system‘s behavior is not determined by the environment and, thus, the degree to which a system determines its own goals”. This definition allows for a potentially quantifiable attribute, rather than an all-or-nothing property. However, it requires some explanation, for example what is meant by ‘goals’ and how a system can be determined by anything other than its environment, given that if it has one, it is not isolated from it. In the main text, I define a ‘goal’ as a fixed point in an objective function, which is a datum that is instantiated in the body of an organism (exemplified by the ‘free energy principle’ [71]). Self determination is a denial of *determinism* (the idea that there is, at any instant, exactly one physically possible future [113,114]), which classical physics takes as axiomatic [14,23]. Any agent, connected to its environment by physical forces must be determined by those forces. Some might suggest spontaneous random events as a way round this—philosophical—problem [123]. True randomness is a deviation from determinism, but is not relevant to organism agency, since by definition the latter must originate from the organism as a system, whereas thermal and quantum fluctuations are exogenous to it. In any biologically meaningful sense, determinism therefore stands for the proposition that all actions of matter can be traced back to an original cause in the abiotic universe [13]. The only escape from this determinist chain is to invoke the stronger condition of *constitutive autonomy*—defined in Froese et al. [27] and discussed by Vernon et al. [61], which is only certain in a system with *causal closure* [14,62,124].
